# District Nursing: II. Some Aspects of the Work

**Published:** 1918-06-22

**Authors:** 


### THE MATRONS' AND SISTERS' DEPARTMENT.

### DISTRICT NURSING.

### II.—Some Aspects of the Work.

Nursing, as a profession, has its drawbacks—some would say it had many—but it has also its points, and amongst them must be reckoned the wide variety of choice of work it offers, making appeal to many different types of people. For keen technical interest, the hospital; for sheer human interest, the district.

Not that these aspects are mutually exclusive, the technical side of district nursing is most important. In no branch of the profession is first-rate work more essential. The poor depend so largely on the nurse, as they cannot afford much doctoring; they tend to be ill oftener, and to have more accidents; the extra work entailed by illness and the loss of earnings often involved presses far more heavily on them. Yes; the poor need better nurses than the rich.

But the district nurse has to be able to do very much more than to nurse the sick-poor; she has to wage everlasting war against the factors that make for sickness and for poverty, and her opportunities are unique. She knows her patients in a very human way, and she does not cease knowing them when they come off the sick list. Bringing the very obvious good of relief in suffering, she gains a large share of gratitude and a definite moral authority. She is a fellow-worker with her patients, but a worker who has a heritage that they have largely been denied and that she can in some measure make good to them.

In hospital the limits of work are strictly defined, but not so on the district; here all the circumstances of the patient form the province of the nurse. In many cases, especially of those "chronics" that always bulk so largely in her list, the actual nursing is almost the least thing she does. It is in the countless opportunities for influence and wise guidance that the real measure of her usefulness lies. To them she is a working woman, working with them (for she generally needs their help), and paid (at least in part) with their subscriptions. So she can mix with them naturally and on level terms, and can exchange notions as from man to man without suspicion of patronage or interference. These "notions" can cover a large field, for there are few of the elements of life that do not react for good or ill on health. Though she is a working woman, it is acknowledged she is "rare knowing and a real scholard," but she does not always find the way easy. She needs a fund of humour, sympathy, and judgment, for the untaught mind is not for that unprejudiced, and the problems of the poor are not written in a book with answers at the end. It is one of the most appealing kinds of work in the world, but it has its dark hours; it is not only when they are grinding vengeance that "the mills of God grind slowly." But she will not go unpaid. The poor may be less wise than the prosperous, but they are not less good, and they continually face the hardness and privation of their lives with a courage and self-sacrifice that is no mean contribution to the spiritual capital of the world and no small inspiration to those who witness it.

The leisure hours of the district nurse and her living arrangements are also very different from those of her hospital sister. Sometimes she is required to live in a central "home," but usually she has to make a little home for herself in lodgings or a tiny flat or cottage. To many this is one of the attractions of district life, especially if friends come often or one perhaps shares the wee home. The common interests of a home make an added bond between the nurse and her women patients, while a garden is one of the surest fields of common interest between her and the men, who will often claim the privilege of doing a "bit of digging" for her "of an evening."

There are drawbacks to the work—wind and weather and irregular hours, perhaps long distances and bad roads. The pay is not princely, and the housing problem is frequently acute. But the work itself has so strong an appeal that it has continually drawn to it some of the very best of the profession, who have found in it a useful and most satisfying life.

☛ The previous article appeared on June 8, p. 209.

